# Diseases of the Urinary Organs

**Published:** 1876-10

**Authors:** 


DISEASES OF THE URINARY ORGANS. A practical treatise on the diseases, injuries, and malformations of the urinary bladder, the prostate gland, and the urethra. By Samuel D. Gross, M.D., LL.D., D.C.L. Oxon, Professor of Surgery in the Jefferson Medical College of Philadelphia. Third edition, revised and edited by Samuel W. Gross, A.M., M.D., Surgeon to the Philadelphia Hospital. Illustrated by one hundred and seventy engravings. Published by Henry C. Lea: Philadelphia. 1876.

For reference and general information, the physician or surgeon can find no work that meets their necessities more thoroughly than this, a revised edition of an excellent treatise, and no medical library should be without it. Replete with handsome illustrations and good ideas, it has the unusual advantage of being easily comprehended, by the reasonable and practical manner in which the various subjects are systematized and arranged. The subjects of stone in the bladder and stricture of the urethra are treated of to an exhaustive extent, and all physicians practicing in that line should possess themselves of the work. We heartily recommend it to the profession as a valuable addition to the important literature of diseases of the urinary organs.

